# Supplementary material for: Supplementing Ryegrass Ameliorates Commercial Diet-Induced Gut Microbial Dysbiosis-Associated Spleen Dysfunctions by Gut–Microbiota–Spleen Axis
Source: Nutrients. 2024 Mar 5;16(5):747. doi: 10.3390/nu16050747 (PMC10935305; doi:10.3390/nu16050747)
Supplement: Supplementary file 1 [file nutrients-16-00747-s001.zip › nutrients-2844354-supplementary/Supplementary information.pdf]

Nutrients

Supplementing Ryegrass Ameliorates Commercial Diet-Induced Gut Microbial Dysbiosis-Associated Spleen Dysfunctions by Gut–Microbiota–Spleen Axis

Qasim Ali [1], Sen Ma [1,2,3], Boshuai Liu [1,2,3], Jiakuan Niu [1], Mengqi Liu [1], Ahsan Mustafa [4], Defeng Li [1,2,3], Zhichang Wang [1,2,3], Hao Sun [1,2,3], Yalei Cui [1,2,3] and Yinghua Shi [1,2,3,*]

[1] Department of Animal Nutrition and Feed Science, College of Animal Science and Technology, Henan
Agricultural University, Zhengzhou 450046, China; qasimali@henau.edu.cn (Q.A.); ms0321021@163.com (S.M.); boshuailiu1@126.com (B.L.); niujiakuan2022@126.com (J.N.); 2019110376@sdau.edu.cn (M.L.); leadephone@126.com (D.L.); zcwang@henau.edu.cn (Z.W.); sunhao@henau.edu.cn (H.S.); yaleicui@henau.edu.cn (Y.C.)

[2] Henan Key Laboratory of Innovation and Utilization of Grassland Resources, Zhengzhou 450002, China

[3] Henan Herbage Engineering Technology Research Center, Zhengzhou 450001, China

[4] Department of Animal Nutrition, Sichuan Agricultural University, Chengdu 611130, China; dr.ahsan.mustafa@gmail.com

* Correspondence: annysyh@henau.edu.cn

Table S1: The nutrients and chemical composition of the diet (two stages) offered to geese.

Ingredients, %	Diets	
	Grower	Finisher
Wheat	57.3	59
Rice bran	5	4
Corn germ cake (exp.)	4	3.2
Corn oil	5	7
Dumpling powder	3	2
Corn distiller’s grains (DDGS)	6.5	7
Spouting germ meal	3	2
Soybean meal (sol.)	7	6
Peanut meal (sol.)	1.5	1

Albumen powder	2	1.5
Stone powder	1.1	1
Liquid methionine	0.25	0.3
MuLaoDa-2	1.25	2
201/202 gunk	2.5	3
Calcium hydrogen phosphate	0.6	1
Chemical composition (%)		
Crude protein	20.12	15.54
Ash	12.89	12.86
Neutral detergent fiber	13.25	30.55
Acid detergent fiber	5.5	27.02
Calcium	1.15	1.07
Phosphorous	0.47	0.32

Table S2. Primer sequences used for quantitative real-time PCR

Gene	Forward primer (Sequence 5′- 3′)	Reverse primer (Sequence 5′- 3′)
Srck	CTGCCGAATCCAAGCTCTTC	TAGTGAGGGAATGAGCCAGC
PI3K	TTCTTGGAGACTGGGAGCAG	GCCTGTTCCTCGTTGCTATG
$P47^{Phox}$	CCAAAGGATGCCAAGAAAAA	CAGCCCCGTTTATTCTTCAA
NADPH	ATGTCCAGCCACCCATGTTA	CAACCCTCCGACGTGATTTC
TLR2	CCTGGTGGTGTCCTTGATCT	GTAGCAGATGTCCTTTGGCG
TLR4	AGGGCTACAGGTCAACAGAC	GACGTTCACCAGCCGAATAC
MyD88	CTGCGTCTTTGATCGGGATG	GGCTTTGCACTTCACTGGAA
NIK	CAACCCTCCGACGTGATTTC	TTTGCTGCTCGATACGCTTC
IKKα	GCATGTACCTCACCCGCTAT	ACACCGACAGGCTTACCATC
NF-κB	GGCAGAGATGGTGGAAGACT	GTTTGCCATCACCACCATGT
IRAK	ACTGCTGAGCTCATGACCTT	TGGGAGAGCACAGTAAGCAC
TRAF6	AGAGGAATGAACTGGCACGA	TTGTCTTACCAGGCGACCTT
TAK1	CGGGATTCAGATGGGCATTG	GCAGTGTCTCAATCGCCATT
IKB-α	AAAGCTGGATGTGACCTGGA	GTTGTAGTTGGTTGCCTGCA
NLRP3	AAACAAACCCGATGCCAAGG	CAGGTTGCTGTGGTTGTTCA
Caspase-1	ATACACTTGCCACGGGAGAC	TGCAAAATCCTTCATGACCA
IL-1β	CACATCACAACCCACAGCAA	CTGCCCCTTCCGTCTTCTTA
IL-18	AAAGAAGGAAGGTGGAGGGG	TCTGTCTCTCTAACGCTGCC
GPR109A	AGCCAGGGGACATATACGTG	TCCCTCGGATCATGAAGCTC
FFAR2	GCACCAGAGATCCTAAGGGG	ATGGGAGGCCTTGACAGAAA
FFAR3	AGCAAGGATCTGGAACTGCT	TCCAAGTACTCAGCAAGGGG
AMPK	TCTCGCCCTCATCCTGAAAG	CTCGACACACTTCAGCCATG
HDAC	GAACAACTTCTGCCACGGTT	CCTCCGACTTCACATCCACT
Keap1	CTGAACGAGGCCCTCAAGTA	CAAACTCGTAGCGGGGAATG
NFE2L2	CGCCTTGAAGCTCATCTCAC	CCTCTCCTGCGTATATCCCG

NRF2	CGCCTTGAAGCTCATCTCAC	CCTCTCCTGCGTATATCCCG
NQ01	GACCCCAAGCACTTCGTCTA	CGAAGCCTTGGATGATGACG
GCLC	GGAGAGTGGAATTCAGGCCT	AGAGCCACATCCATCCACAA
GCLM	TGCCCCACCTCCTATTGAAG	TGTGAGATCAGGTGGCATCA
GSTA4	GCACCTCTGTTCCAAAGCAA	AATCCAGGACACAGCCATCA
HMOX1	AGGGCTGGCTTTCTTTACCT	GTGACCGTTTTCCTGGCTTT
SOD	GGAAGAATCAGCGGCTTGTC	CCAATGATGCAGTGTGGTCC
GPx	GCTTCCCCTCCAACCAATTC	TAGACCTTCTGCTCCTTGGC
CAT	CCGAGTCTTTGCATCAGGTG	GCCTTCCTGCTTCCTCTACA
IL-4	TGACAGGGTATTGGTCCACC	ACGGAAGAAGCAGAAGGTGA
IL-10	CATCAAGAACAGCGAGCACC	GCACCCACCTTTTCAAACGT
iNOS	CTCATTCTCCAAGCGAACGG	GCACTCCTATCTCTGTCCCC
COX2	GGTTCTACAATGGAGAGCGC	TGTTCTTGCCACTTGAGCTG
TNF-α	GGTCCACAACGAGTTCATCC	AGGAGGAGGAGGAGATGGAG
IL-6	GGAAGACCCTTGCTCTCCTT	TGGAGCCAGAAGATGAGTGG
CD4	AGCAAGACAGAGCTGGTGGT	TCCATCAGGTGACAGTTCCA
B220	GGCCATACTACCCAAAGCAA	ATGCCTCCTGCTTGGTTTTT
CD19	GGAGGAGAATTGGATGCTGA	CTCGTTCTCCACTCGTAGCC
IgD	TTTCTAACAGCACCGCCTCT	GGTCGCTCTTCGTCTTCATC
CD3	TTGGGTGCAATTTATGACGA	ACAGCAACCATCAGGAGGAC
CD8	GTGAAGTTGCCACAGCAAAA	GCAGCTTTTTGGAGAGGATG
IL-2	AGAAAAGCTGGGAACAAGCA	ACAGATCTTGCATCCCGTTC
TGF-B	AGCTAGCGGTCTTGCACATT	GATTGTTCCCCACATCCAAC
IL-17	CTGCCTACGGGAAGGTGATA	GGTCCTCGTCGATCCTGTAA
β-actin	CAACGAGCGGTTCAGGTGT	TGGAGTTGAAGGTGGTCTCG

**Species**	**Relative abundance in IHF group (45 Days)**	**Relative abundance in AGF group (45 Days)**	**Log2FC**	***P*-Value**	**FDR**
Clostridia	55.7666667	32.5500000	-0.7767455	1.06E-06	1.32E-06
Bacteroidia	43.5566667	20.5766667	-1.0818843	9.47E-08	1.70E-07
Bacilli	11.5700000	12.5433333	0.1165319	5.86E-06	6.21E-06
Coriobacteriia	3.4076667	3.5986667	0.0786783	1.37E-05	1.37E-05
Actinobacteria	2.7770000	5.2946667	0.9310127	3.51E-08	9.46E-08
Vampirivibrionia	1.2173333	4.9043333	2.0103328	1.47E-07	2.52E-07

Gammaproteobacteria	0.3723333	0.1938667	-0.9415300	6.98E-08	1.40E-07
Desulfovibrionia	0.3084333	0.2547667	-0.2757822	4.82E-06	5.26E-06
Verrucomicrobiae	0.0062670	0.2127667	5.0853533	5.06E-08	1.07E-07
Alphaproteobacteria	0.0092160	0.4268333	5.5333883	3.00E-09	1.08E-08
Negativicutes	0.1705000	0.0766767	-1.1529122	2.12E-06	2.39E-06
**Species**	**Relative abundance in IHF group (60 Days)**	**Relative abundance in AGF group (60 Days)**	**Log2FC**	***P*-Value**	**FDR**
Clostridia	58.7833333	47.9333333	-0.2943779	3.82E-06	3.82E-06
Bacteroidia	17.6533333	16.0700000	-0.1355707	2.22E-06	2.28E-06
Bacilli	9.1833333	25.5633333	1.4769862	1.86E-07	3.53E-07
Coriobacteriia	8.3870000	6.4136667	-0.3870055	3.57E-07	5.35E-07
Actinobacteria	0.1537333	1.5070000	3.2931775	2.55E-07	4.59E-07
Vampirivibrionia	0.4473667	2.2076667	2.3029927	2.58E-08	7.15E-08
Gammaproteobacteria	2.9070000	1.0683333	-1.4441692	6.57E-08	1.48E-07
Desulfovibrionia	0.5774333	0.1263667	-2.1920384	2.73E-09	1.09E-08
Verrucomicrobiae	0.1465000	0.2055000	0.4882377	3.17E-09	1.74E-08
Alphaproteobacteria	0.0309700	0.0390767	0.3354360	8.45E-08	1.69E-07
Negativicutes	0.0313500	0.0140367	-1.1592651	7.41E-08	1.57E-07
**Species**	**Relative abundance in IHF group (90 Days)**	**Relative abundance in AGF group (90 Days)**	**Log2FC**	***P*-Value**	**FDR**
Clostridia	61.4633333	46.6433333	-0.3980551	9.11E-07	1.21E-06
Bacteroidia	32.6400000	14.6600000	-1.1547560	1.55E-07	2.78E-07
Bacilli	10.2233333	14.4833333	0.5025280	2.48E-05	2.63E-05
Coriobacteriia	5.2470000	6.3533333	0.2760209	7.26E-07	1.03E-06
Actinobacteria	0.2707000	1.2470000	2.2036947	4.25E-07	6.95E-07
Vampirivibrionia	0.3247000	0.9645000	1.5706739	9.18E-02	5.21E-08
Gammaproteobacteria	0.1896333	0.0733633	-1.3700815	5.33E-07	8.34E-07
Desulfovibrionia	1.8086667	1.7050000	-0.0851548	2.53E-04	2.60E-04
Verrucomicrobiae	0.0910533	0.1166333	0.3571964	1.10E-05	1.24E-05
Alphaproteobacteria	0.0427700	0.1185000	1.4702159	3.15E-06	3.78E-06
Negativicutes	0.1457000	0.0785367	-0.8915626	5.96E-07	8.94E-07

Table S3. Effect of ryegrass on gut microbial richness of geese at class level

Table S4. Effect of ryegrass on the gut microbial richness of geese at a family level

Species	Relative abundance in IHF group (45 Days)	Relative abundance in AGF group (45 Days)	Log2FC	*P*-Value	FDR
Ruminococcaceae	22.133333	8.607	-1.362638	4.16E-07	5.71E-07
Bacteroidaceae	8.884333	23.143333	1.381261	1.19E-06	1.44E-06
Lachnospiraceae	9.985333	8.288333	-0.268729	6.94E-07	8.96E-07
norank_o__Clostridia_UCG-014	8.146	0.989767	-3.040931	8.51E-09	5.67E-08
Oscillospiraceae	4.448333	6.825667	0.617705	1.57E-07	3.31E-07
Rikenellaceae	3.736667	8.908333	1.253404	8.31E-09	5.67E-08
norank_o__RF39	3.033667	0.1326	-4.51591	1.38E-07	3.06E-07
Prevotellaceae	5.265667	3.224333	-0.707615	3.33E-07	4.94E-07
Eubacterium_coprostanoligenes_group	3.375	0.062727	-5.749665	3.97E-08	1.36E-07
Erysipelatoclostridiaceae	4.366667	1.179	-1.888969	2.19E-06	2.57E-06
Enterococcaceae	3.155	0.153766667	-4.358825	2.07E-07	3.95E-07
Eggerthellaceae	1.498333	2.818667	0.911654	3.19E-08	1.36E-07
Peptococcaceae	2.227333	1.647667	-0.434893	2.65E-06	3.02E-06
Christensenellaceae	1.448333	0.6197	-1.224752	4.28E-07	5.71E-07
Lactobacillaceae	0.697533	6.156667	3.141815	4.59E-06	4.96E-06
Peptostreptococcaceae	1.457667	0.02545	-5.839851	1.02E-07	2.62E-07
norank_o__Gastranaerophilales	1.216667	4.904333	2.011123	2.61E-07	4.36E-07
Erysipelotrichaceae	4.4	0.254767	-4.110255	1.16E-03	1.16E-03
Bifidobacteriaceae	1.834667	5.258333	1.519088	2.32E-07	4.06E-07
Butyricicoccaceae	1.083333	2.337667	1.109592	8.83E-07	1.10E-06
Coriobacteriaceae	0.969533	0.126833	-2.934357	7.04E-09	5.67E-08
Tannerellaceae	0.6593	3.343667	2.342424	4.22E-07	5.71E-07
Muribaculaceae	0.795333	2.786667	1.808909	2.75E-06	3.05E-06
Atopobiaceae	1.133333	0.001856	-9.253901	2.12E-04	2.18E-04
Anaerovoracaceae	0.553667	0.189333	-1.548089	1.05E-07	2.62E-07
Desulfovibrionaceae	0.254667	0.3084	0.276193	9.43E-06	9.92E-06
norank_o__Bacteroidales	0.038767	0.8877	4.517184	7.71E-10	2.67E-08
Barnesiellaceae	1.838667	0.3237	-2.505931	4.09E-08	1.36E-07
Streptococcaceae	0.063767	0.339567	2.41282	3.22E-08	1.36E-07
Enterobacteriaceae	0.3676	0.099543	-1.88474	4.09E-08	1.36E-07
norank_o__Clostridia_vadinBB60_group	0.049383	2.185667	5.467905	3.07E-07	4.91E-07
Coriobacteriales_Incertae_Sedis	0.020273	0.459467	4.502305	6.92E-08	1.98E-07
Monoglobaceae	0.085883	0.220733	1.361854	1.35E-07	3.06E-07
UCG-010	0.2047	0.399867	0.966008	2.33E-07	4.06E-07
Succinivibrionaceae	0.022477	0.001107	-4.343701	2.06E-08	1.17E-07
Planococcaceae	0.000369	0.003318	3.168737	6.39E-09	5.67E-08
Akkermansiaceae	0.006267	0.212767	5.085353	5.06E-08	1.56E-07
Marinifilaceae	0.134833	0.019177	-2.813753	2.01E-07	3.95E-07

Anaerofustaceae	0.2925	0.050877	-2.523361	3.20E-07	4.92E-07
unclassified_c__Clostridia	0.026177	0.090683	1.792556	1.34E-09	2.67E-08
**Species**	**Relative abundance in IHF group (60 Days)**	**Relative abundance in AGF group (60 Days)**	**Log2FC**	***P*-Value**	**FDR**
Ruminococcaceae	21.606667	10.543333	-1.035145	1.82E-07	4.84E-07
Bacteroidaceae	6.536	8.072	0.304518	2.12E-07	5.02E-07
Lachnospiraceae	3.97	14.536667	1.872486	1.24E-06	1.99E-06
norank_o__Clostridia_UCG-014	11.846667	4.994667	-1.246021	3.83E-07	8.52E-07
Oscillospiraceae	3.095333	4.543667	0.553762	1.30E-06	2.00E-06
Rikenellaceae	3.9733333	4.427	0.155980	0.022095	0.022095
norank_o__RF39	6.394667	3.058	-1.064281	1.25E-07	4.49E-07
Prevotellaceae	4.373	0.9485	-2.204904	1.54E-07	4.49E-07
Eubacterium_coprostanoligenes_group	7.504	2.85	-1.396698	1.87E-06	2.58E-06
Erysipelatoclostridiaceae	2.615667	2.35	-0.154518	6.16E-04	6.32E-04
Enterococcaceae	9.964333	1.706	-2.546156	3.26E-09	3.26E-08
Eggerthellaceae	2.533333	4.263333	0.750945	1.67E-05	1.86E-05
Peptococcaceae	5.593667	0.477433	-3.550423	4.49E-08	2.00E-07
Christensenellaceae	2.386333	1.484333	-0.68498	2.46E-06	3.17E-06
Lactobacillaceae	0.135667	3.667	4.756462	1.78E-10	3.56E-09
Peptostreptococcaceae	2.004333	4.556	1.184645	2.13E-07	5.02E-07
norank_o__Gastranaerophilales	0.4473	2.206333	2.302336	1.21E-07	4.49E-07
Erysipelotrichaceae	1.294667	1.924333	0.571778	5.60E-07	1.13E-06
Bifidobacteriaceae	0.04348	1.463333	5.072763	2.77E-06	3.47E-06
Butyricicoccaceae	0.434833	0.864633	0.991626	2.99E-24	1.19E-22
Coriobacteriaceae	4.105	0.1717	-4.57942	3.91E-08	1.96E-07
Tannerellaceae	0.584633	0.498433	-0.230132	6.73E-07	1.24E-06
Muribaculaceae	0.407333	0.5825	0.516048	9.58E-06	1.09E-05
Atopobiaceae	1.493667	0.548733	-1.444681	1.68E-06	2.40E-06
Anaerovoracaceae	1.087333	0.611567	-0.830213	1.57E-07	4.49E-07
Desulfovibrionaceae	0.126267	0.577333	2.192931	2.73E-09	3.26E-08
norank_o__Bacteroidales	0.714633	1.056667	0.564245	2.30E-06	3.06E-06
Barnesiellaceae	0.711767	0.556367	-0.355368	8.28E-07	1.44E-06
Streptococcaceae	0.290733	0.364333	0.325563	3.69E-06	4.48E-06
Enterobacteriaceae	2.786333	0.085873	-5.020014	7.93E-09	5.69E-08
norank_o__Clostridia_vadinBB60_group	0.126533	0.222567	0.81472	1.51E-06	2.23E-06
Coriobacteriales_Incertae_Sedis	0.212667	1.185	2.478221	5.65E-07	1.13E-06
Monoglobaceae	0.173	0.295567	0.772712	1.38E-04	1.45E-04
UCG-010	0.096567	0.225367	1.222677	8.71E-07	1.45E-06
Succinivibrionaceae	0.9567	0.037667	-4.666706	1.05E-08	6.01E-08
Planococcaceae	0.003676	0.590567	7.327819	8.53E-09	5.69E-08
Akkermansiaceae	0.145367	0.205433	0.498974	1.54E-07	4.49E-07
Marinifilaceae	0.226667	0.102267	-1.148236	8.08E-06	9.50E-06

Anaerofustaceae	0.06708	0.056533	-0.246781	9.23E-05	9.98E-05
unclassified_c__Clostridia	0.05346	0.064867	0.279017	6.83E-07	1.24E-06
**Species**	**Relative abundance in IHF group (90 Days)**	**Relative abundance in AGF group (90 Days)**	**Log2FC**	***P*-Value**	**FDR**
Ruminococcaceae	12.583333	18.356667	0.54479	1.67E-07	5.77E-07
Bacteroidaceae	6.726333	14.38	1.096171	9.11E-07	1.92E-06
Lachnospiraceae	9.352667	9.525667	0.026442	1.67E-05	1.91E-05
norank_o__Clostridia_UCG-014	10.746667	9.762333	-0.138591	1.12E-05	1.36E-05
Oscillospiraceae	5.114	10.18	0.993213	2.36E-06	3.49E-06
Rikenellaceae	3.644667	8.455	1.2140179	9.6E-09	1.28E-07
norank_o__RF39	5.885	4.733333	-0.314186	0.000394	0.000404
Prevotellaceae	5.132667	1.594333	-1.686755	4.44E-09	1.28E-07
Eubacterium_coprostanoligenes_group	2.935333	1.236333	-1.247457	1.73E-07	5.77E-07
Erysipelatoclostridiaceae	4.263	2.213667	-0.945431	1.32E-08	1.32E-07
Enterococcaceae	1.398333	0.133333	-3.390599	1.25E-06	2.3E-06
Eggerthellaceae	1.002667	3.946667	1.976793	0.000013	1.53E-05
Peptococcaceae	0.861733	0.648767	-0.409542	1.96E-07	5.81E-07
Christensenellaceae	3.198333	1.894333	-0.75563	1.18E-06	2.3E-06
Lactobacillaceae	0.1267	0.155533	0.295807	1.07E-05	1.34E-05
Peptostreptococcaceae	1.406667	0.683733	-1.040775	6.8E-08	3.02E-07
norank_o__Gastranaerophilales	0.964433	0.324633	-1.57087	4.89E-08	2.44E-07
Erysipelotrichaceae	1.445333	0.907267	-0.671804	2.35E-06	3.49E-06
Bifidobacteriaceae	0.098057	1.237333	3.657475	4.11E-08	2.35E-07
Butyricicoccaceae	1.074	2.835667	1.400694	2.86E-07	7.64E-07
Coriobacteriaceae	1.785	0.817667	-1.126339	4.65E-07	1.1E-06
Tannerellaceae	1.267667	0.4507	-1.491936	4.08E-08	2.35E-07
Muribaculaceae	0.660567	1.287667	0.962983	2.03E-07	5.81E-07
Atopobiaceae	2.124333	1.193	-0.832416	1.6E-06	2.67E-06
Anaerovoracaceae	1.846667	1.125	-0.714998	1.71E-06	2.73E-06
Desulfovibrionaceae	1.704333	1.808	0.085187	0.000253	0.000266
norank_o__Bacteroidales	0.9415	0.949467	0.012156	0.00014	0.000151
Barnesiellaceae	0.439633	0.3577	-0.297551	1.32E-06	2.3E-06
Streptococcaceae	0.8545	1.828	1.097114	6.38E-07	1.42E-06
Enterobacteriaceae	0.065623	0.005528	-3.569379	2.84E-08	2.28E-07
norank_o__Clostridia_vadinBB60_group	0.151833	0.3928	1.371306	9.57E-09	1.28E-07
Coriobacteriales_Incertae_Sedis	0.254433	0.283333	0.155213	5.99E-06	7.98E-06
Monoglobaceae	0.3748	0.4634	0.306137	2.55E-06	3.64E-06
UCG-010	0.183833	0.204567	0.154173	1.29E-06	2.3E-06
Succinivibrionaceae	0.03983	0.024667	-0.691293	6.19E-06	7.98E-06
Planococcaceae	0.004056	0.257333	5.987437	3.47E-06	4.79E-06
Akkermansiaceae	0.09105	0.1165	0.355599	7.72E-08	3.09E-07
Marinifilaceae	0.125667	0.07115	-0.820666	3.16E-07	7.9E-07

Anaerofustaceae	0.04903	0.038633	-0.343819	2.72E-05	3.02E-05
unclassified_c__Clostridia	0.1464	0.148467	0.020224	0.00207	0.00207

Table S5. Gut microbiota and KEGG orthologues involved in LPS production

**Family**	**KEGG strains regarding LPS biosynthesis**	**Correlation**	***P*-value**	**Adj *P*-value**	**Significance**
Ruminococcaceae	Bacterial invasion of epithelial cells	0.7812	0.0001	0.0005	***
Ruminococcaceae	Legionellosis	0.4550	0.0578	0.1386	
Ruminococcaceae	Epithelial cell signaling in Helicobacter pylori infection	0.2321	0.3541	0.6912	
Ruminococcaceae	Salmonella infection	0.2922	0.2393	0.4102	
Ruminococcaceae	Pertussis	0.6236	0.0057	0.0091	**
Ruminococcaceae	Vibrio cholerae infection	0.1813	0.4715	0.6708	
Bacteroidaceae	Bacterial invasion of epithelial cells	-0.6212	0.0059	0.0071	**
Bacteroidaceae	Legionellosis	-0.2588	0.2998	0.4498	
Bacteroidaceae	Epithelial cell signaling in Helicobacter pylori infection	-0.4810	0.0433	0.1299	*
Bacteroidaceae	Salmonella infection	-0.3650	0.1364	0.3274	
Bacteroidaceae	Pertussis	-0.8782	0.0000	0.0000	***
Bacteroidaceae	Vibrio cholerae infection	-0.5081	0.0313	0.0752	*
Lachnospiraceae	Bacterial invasion of epithelial cells	-0.5630	0.0150	0.0163	*
Lachnospiraceae	Legionellosis	-0.1201	0.6349	0.7619	
Lachnospiraceae	Epithelial cell signaling in Helicobacter pylori infection	0.0865	0.7328	0.8564	
Lachnospiraceae	Salmonella infection	-0.2964	0.2324	0.4102	
Lachnospiraceae	Pertussis	-0.2175	0.3859	0.3859	
Lachnospiraceae	Vibrio cholerae infection	-0.0442	0.8618	0.8618	
norank_o__Clostridia_UCG-014	Bacterial invasion of epithelial cells	0.6676	0.0025	0.0037	**
norank_o__Clostridia_UCG-014	Legionellosis	0.4894	0.0393	0.1178	*
norank_o__Clostridia_UCG-014	Epithelial cell signaling in Helicobacter pylori infection	0.7280	0.0006	0.0037	***
norank_o__Clostridia_UCG-014	Salmonella infection	0.0239	0.9251	0.9251	
norank_o__Clostridia_UCG-014	Pertussis	0.6260	0.0054	0.0091	**
norank_o__Clostridia_UCG-014	Vibrio cholerae infection	0.1205	0.6338	0.7481	
Oscillospiraceae	Bacterial invasion of epithelial cells	-0.6302	0.0051	0.0067	**
Oscillospiraceae	Legionellosis	0.0890	0.7253	0.7913	
Oscillospiraceae	Epithelial cell signaling in Helicobacter pylori infection	0.3306	0.1803	0.4327	
Oscillospiraceae	Salmonella infection	-0.8666	0.0000	0.0000	***
Oscillospiraceae	Pertussis	-0.8019	0.0001	0.0002	***
Oscillospiraceae	Vibrio cholerae infection	-0.7021	0.0012	0.0046	**
Rikenellaceae	Bacterial invasion of epithelial cells	-0.7054	0.0011	0.0023	**

Rikenellaceae	Legionellosis	-0.2827	0.2556	0.4382	
Rikenellaceae	Epithelial cell signaling in Helicobacter pylori infection	-0.2038	0.4173	0.6912	
Rikenellaceae	Salmonella infection	-0.5458	0.0191	0.0573	*
Rikenellaceae	Pertussis	-0.8866	0.0000	0.0000	***
Rikenellaceae	Vibrio cholerae infection	-0.5117	0.0299	0.0752	*
norank_o__RF39	Bacterial invasion of epithelial cells	0.5459	0.0191	0.0191	*
norank_o__RF39	Legionellosis	0.3057	0.2173	0.4347	
norank_o__RF39	Epithelial cell signaling in Helicobacter pylori infection	0.7277	0.0006	0.0037	***
norank_o__RF39	Salmonella infection	0.0469	0.8534	0.9251	
norank_o__RF39	Pertussis	0.6198	0.0061	0.0091	**
norank_o__RF39	Vibrio cholerae infection	0.2324	0.3533	0.6057	
Prevotellaceae	Bacterial invasion of epithelial cells	0.8187	0.0000	0.0004	***
Prevotellaceae	Legionellosis	0.6896	0.0015	0.0062	**
Prevotellaceae	Epithelial cell signaling in Helicobacter pylori infection	0.0488	0.8474	0.8564	
Prevotellaceae	Salmonella infection	0.2666	0.2849	0.4274	
Prevotellaceae	Pertussis	0.3968	0.1030	0.1236	
Prevotellaceae	Vibrio cholerae infection	-0.1688	0.5031	0.6708	
Eubacterium_coprostanoligenes_group	Bacterial invasion of epithelial cells	0.7803	0.0001	0.0005	***
Eubacterium_coprostanoligenes_group	Legionellosis	-0.0268	0.9160	0.9160	
Eubacterium_coprostanoligenes_group	Epithelial cell signaling in Helicobacter pylori infection	0.0459	0.8564	0.8564	
Eubacterium_coprostanoligenes_group	Salmonella infection	0.7393	0.0005	0.0018	***
Eubacterium_coprostanoligenes_group	Pertussis	0.9282	0.0000	0.0000	***
Eubacterium_coprostanoligenes_group	Vibrio cholerae infection	0.7297	0.0006	0.0045	***
Erysipelatoclostridiaceae	Bacterial invasion of epithelial cells	0.6954	0.0014	0.0023	**
Erysipelatoclostridiaceae	Legionellosis	0.8117	0.0000	0.0005	***
Erysipelatoclostridiaceae	Epithelial cell signaling in Helicobacter pylori infection	0.5156	0.0285	0.1141	*
Erysipelatoclostridiaceae	Salmonella infection	0.0375	0.8826	0.9251	
Erysipelatoclostridiaceae	Pertussis	0.5640	0.0148	0.0197	*
Erysipelatoclostridiaceae	Vibrio cholerae infection	-0.1025	0.6858	0.7481	
Enterococcaceae	Bacterial invasion of epithelial cells	0.7481	0.0004	0.0011	***
Enterococcaceae	Legionellosis	-0.1335	0.5976	0.7619	
Enterococcaceae	Epithelial cell signaling in Helicobacter pylori infection	-0.1629	0.5184	0.6912	
Enterococcaceae	Salmonella infection	0.8053	0.0001	0.0003	***
Enterococcaceae	Pertussis	0.8240	0.0000	0.0001	***
Enterococcaceae	Vibrio cholerae infection	0.7205	0.0007	0.0045	***
Eggerthellaceae	Bacterial invasion of epithelial cells	-0.6965	0.0013	0.0023	**
Eggerthellaceae	Legionellosis	-0.7564	0.0003	0.0017	***

Eggerthellaceae	Epithelial cell signaling in Helicobacter pylori infection	-0.1672	0.5072	0.6912	
Eggerthellaceae	Salmonella infection	-0.0952	0.7071	0.9251	
Eggerthellaceae	Pertussis	-0.2843	0.2528	0.2758	
Eggerthellaceae	Vibrio cholerae infection	0.3031	0.2215	0.4430	

Table S6. Effect of ryegrass on the relationship of gut microbiota at class level and SCFAs

Class	SCFAs	Correlation	*P*-value	Adj *P*-value	Significance
Clostridia	Acetic acid	-0.760860651	0.000245813	0.000807587	***
Clostridia	Propionic acid	-0.596365112	0.008993928	0.019588471	**
Clostridia	Butyric acid	-0.275107922	0.269210243	0.423044667	
Clostridia	Isovaleric acid	-0.616862556	0.006393533	0.011721477	**
Clostridia	Valeric acid	-0.743142063	0.000409572	0.001501762	***
Bacteroidia	Acetic acid	0.747065026	0.000367085	0.000807587	***
Bacteroidia	Propionic acid	0.585489825	0.010684621	0.019588471	*
Bacteroidia	Butyric acid	0.382701362	0.117006009	0.214511016	
Bacteroidia	Isovaleric acid	0.660509643	0.002847679	0.006264895	**
Bacteroidia	Valeric acid	0.690451586	0.001514367	0.004164508	**
Bacilli	Acetic acid	-0.059546753	0.81443691	0.81443691	
Bacilli	Propionic acid	-0.042888517	0.865816902	0.865816902	
Bacilli	Butyric acid	-0.137166979	0.587303383	0.646033722	
Bacilli	Isovaleric acid	-0.225329295	0.368652895	0.368652895	
Bacilli	Valeric acid	-0.027773637	0.91288959	0.91288959	
Coriobacteriia	Acetic acid	-0.750289603	0.000334995	0.000807587	***
Coriobacteriia	Propionic acid	-0.615231128	0.006575051	0.018081389	**
Coriobacteriia	Butyric acid	-0.535070837	0.022130606	0.060859168	*
Coriobacteriia	Isovaleric acid	-0.804710516	5.65E-05	0.000621147	***
Coriobacteriia	Valeric acid	-0.547085879	0.018787949	0.034444574	*
Actinobacteria	Acetic acid	0.282118009	0.256699181	0.352961374	
Actinobacteria	Propionic acid	0.205654663	0.412965663	0.567827787	
Actinobacteria	Butyric acid	0.105539165	0.676834527	0.676834527	
Actinobacteria	Isovaleric acid	0.675201286	0.002107151	0.005794665	**
Actinobacteria	Valeric acid	0.141273895	0.5760514	0.704062822	
Vampirivibrionia	Acetic acid	0.858516074	5.13E-06	2.82E-05	***
Vampirivibrionia	Propionic acid	0.752317227	0.000316052	0.001738285	***
Vampirivibrionia	Butyric acid	0.39450113	0.105217636	0.214511016	
Vampirivibrionia	Isovaleric acid	0.775033003	0.000158301	0.000870656	***
Vampirivibrionia	Valeric acid	0.848006077	8.79E-06	4.83E-05	***
Gammaproteobacteria	Acetic acid	-0.584250244	0.010892466	0.017116733	*
Gammaproteobacteria	Propionic acid	-0.495291065	0.036618501	0.057543358	*
Gammaproteobacteria	Butyric acid	-0.649178796	0.003554555	0.01955005	**

Gammaproteobacteria	Isovaleric acid	-0.38964613	0.109960148	0.134395736	
Gammaproteobacteria	Valeric acid	-0.367365434	0.133685619	0.210077401	
Desulfovibrionia	Acetic acid	-0.204855733	0.414822928	0.507005801	
Desulfovibrionia	Propionic acid	-0.178886422	0.477564086	0.583689439	
Desulfovibrionia	Butyric acid	0.218446909	0.383839731	0.469137449	
Desulfovibrionia	Isovaleric acid	-0.463484786	0.052716169	0.082839694	
Desulfovibrionia	Valeric acid	-0.262926382	0.291832761	0.401270046	
Verrucomicrobiae	Acetic acid	-0.674859318	0.002122366	0.003891004	**
Verrucomicrobiae	Propionic acid	-0.625309366	0.005517479	0.018081389	**
Verrucomicrobiae	Butyric acid	-0.56139749	0.015340034	0.05624679	*
Verrucomicrobiae	Isovaleric acid	-0.442374823	0.066021251	0.09077922	
Verrucomicrobiae	Valeric acid	-0.675302275	0.002102675	0.004625885	**
Alphaproteobacteria	Acetic acid	0.895861132	0	5.49E-06	***
Alphaproteobacteria	Propionic acid	0.866090727	3.39E-06	3.73E-05	***
Alphaproteobacteria	Butyric acid	0.661119518	0.002813183	0.01955005	**
Alphaproteobacteria	Isovaleric acid	0.743842552	0.000401696	0.001472886	***
Alphaproteobacteria	Valeric acid	0.888353753	8.51E-07	9.36E-06	***
Negativicutes	Acetic acid	0.177021289	0.482242506	0.530466756	
Negativicutes	Propionic acid	0.05958413	0.814322352	0.865816902	
Negativicutes	Butyric acid	0.245332174	0.326475723	0.448904119	
Negativicutes	Isovaleric acid	0.298804715	0.228408411	0.251249252	
Negativicutes	Valeric acid	-0.058013169	0.81914022	0.901054242	

Table S7. Effect of ryegrass on the relationship of gut microbiota at a family level and SCFAs

**Family**	**SCFAs**	**Correlation**	***P*-value**	**Adj *P*-value**	**Significance**
Ruminococcaceae	Acetic acid	-0.5828454	0.0111319	0.0190833	*
Ruminococcaceae	Propionic acid	-0.4575359	0.0562406	0.0749875	
Ruminococcaceae	Butyric acid	-0.2415943	0.3341332	0.445511	
Ruminococcaceae	Isovaleric acid	-0.2423201	0.3326381	0.4989571	
Ruminococcaceae	Valeric acid	-0.5956671	0.0090955	0.0155923	**
Bacteroidaceae	Acetic acid	0.91292962	1.26E-07	1.51E-06	***
Bacteroidaceae	Propionic acid	0.92041418	6.29E-08	7.55E-07	***
Bacteroidaceae	Butyric acid	0.79392723	8.37E-05	0.0003418	***
Bacteroidaceae	Isovaleric acid	0.76442845	0.0002207	0.0013239	***
Bacteroidaceae	Valeric acid	0.86549546	3.50E-06	4.20E-05	***
Lachnospiraceae	Acetic acid	0.12276315	0.6274701	0.6845128	
Lachnospiraceae	Propionic acid	0.06583131	0.7952275	0.7952275	
Lachnospiraceae	Butyric acid	0.05964273	0.8141428	0.8141428	
Lachnospiraceae	Isovaleric acid	-0.0583754	0.8180288	0.8180288	
Lachnospiraceae	Valeric acid	0.0345728	0.8916715	0.8916715	

norank_o__Clostridia_UCG-014	Acetic acid	-0.8330951	1.77E-05	7.07E-05	***
norank_o__Clostridia_UCG-014	Propionic acid	-0.7583392	0.000265	0.0007274	***
norank_o__Clostridia_UCG-014	Butyric acid	-0.4358307	0.0706124	0.1210499	
norank_o__Clostridia_UCG-014	Isovaleric acid	-0.7341886	0.0005222	0.0020889	***
norank_o__Clostridia_UCG-014	Valeric acid	-0.8034709	5.92E-05	0.0003549	***
Oscillospiraceae	Acetic acid	0.43131856	0.0739127	0.0985503	
Oscillospiraceae	Propionic acid	0.53125422	0.0232846	0.0349269	*
Oscillospiraceae	Butyric acid	0.7933386	8.55E-05	0.0003418	***
Oscillospiraceae	Isovaleric acid	0.13405773	0.5958825	0.6500536	
Oscillospiraceae	Valeric acid	0.33763408	0.1706049	0.2274731	
Rikenellaceae	Acetic acid	0.72096771	0.0007352	0.0017645	***
Rikenellaceae	Propionic acid	0.80096213	6.49E-05	0.0002597	***
Rikenellaceae	Butyric acid	0.81743235	3.44E-05	0.0003418	***
Rikenellaceae	Isovaleric acid	0.47868561	0.0444666	0.1067198	*
Rikenellaceae	Valeric acid	0.69710176	0.0013032	0.0039097	**
norank_o__RF39	Acetic acid	-0.8660976	3.39E-06	2.03E-05	***
norank_o__RF39	Propionic acid	-0.8011408	6.45E-05	0.0002597	***
norank_o__RF39	Butyric acid	-0.5274954	0.0244666	0.0489331	*
norank_o__RF39	Isovaleric acid	-0.8504904	7.77E-06	9.32E-05	***
norank_o__RF39	Valeric acid	-0.7900829	9.58E-05	0.0003831	***
Prevotellaceae	Acetic acid	-0.1293386	0.6090011	0.6845128	
Prevotellaceae	Propionic acid	-0.2398059	0.3378335	0.4054002	
Prevotellaceae	Butyric acid	-0.2229378	0.3738913	0.4486696	
Prevotellaceae	Isovaleric acid	0.17956409	0.4758698	0.6344931	
Prevotellaceae	Valeric acid	-0.2038791	0.4170993	0.5005192	
Eubacterium_coprostanoligenes_group	Acetic acid	-0.7999961	6.73E-05	0.0002018	***
Eubacterium_coprostanoligenes_group	Propionic acid	-0.7537672	0.0003031	0.0007274	***
Eubacterium_coprostanoligenes_group	Butyric acid	-0.7827986	0.0001227	0.0003682	***
Eubacterium_coprostanoligenes_group	Isovaleric acid	-0.5255044	0.0251114	0.0753341	*
Eubacterium_coprostanoligenes_group	Valeric acid	-0.6525184	0.0033327	0.0079986	**
Erysipelatoclostridiaceae	Acetic acid	-0.5100922	0.0305622	0.0458432	*
Erysipelatoclostridiaceae	Propionic acid	-0.598447	0.0086963	0.0173926	**
Erysipelatoclostridiaceae	Butyric acid	-0.3963547	0.1034463	0.1551694	
Erysipelatoclostridiaceae	Isovaleric acid	-0.2963813	0.2323871	0.3983778	
Erysipelatoclostridiaceae	Valeric acid	-0.6332507	0.0047854	0.0095707	**
Enterococcaceae	Acetic acid	-0.6304261	0.0050361	0.0100722	**
Enterococcaceae	Propionic acid	-0.5686779	0.0137901	0.0236402	*
Enterococcaceae	Butyric acid	-0.6642466	0.0026417	0.00634	**
Enterococcaceae	Isovaleric acid	-0.3278104	0.1841826	0.3683652	
Enterococcaceae	Valeric acid	-0.4672694	0.0505622	0.0758434	
Eggerthellaceae	Acetic acid	0.07446043	0.7690354	0.7690354	
Eggerthellaceae	Propionic acid	0.21938272	0.3817547	0.4164597	

Eggerthellaceae	Butyric acid	0.17075289	0.498129	0.5434135	
Eggerthellaceae	Isovaleric acid	-0.1544552	0.5405751	0.6486901	
Eggerthellaceae	Valeric acid	0.17964852	0.4756589	0.5189007	

Table S8: Effect of ryegrass on the relationship of gut microbiota and host markers

Family	Host markers	Correlation	*P*-value
Ruminococcaceae	NLRP3	0.822088731	2.84E-05
norank_o__Clostridia_UCG-014	T-AOC	-0.87738917	1.74E-06
norank_o__Clostridia_UCG-014	CAT	-0.816130055	3.62E-05
Oscillospiraceae	IgM	0.921699728	5.55E-08
Oscillospiraceae	IgD	0.828379199	2.18E-05
Oscillospiraceae	MDA	-0.824228115	2.60E-05
Oscillospiraceae	NFE2L2	0.93727161	9.89E-09
Oscillospiraceae	B220	0.855771889	5.93E-06
Oscillospiraceae	CD8	0.933653344	1.53E-08
Oscillospiraceae	CD3	0.964208136	1.21E-10
Rikenellaceae	GSR	0.828623167	2.15E-05
Rikenellaceae	NFE2L2	0.865444978	3.51E-06
Rikenellaceae	CD3	0.879847477	1.49E-06
norank_o__RF39	T-AOC	-0.822880024	2.75E-05
norank_o__RF39	HDAC	0.800443762	6.62E-05
Prevotellaceae	IgG	-0.897932876	4.28E-07
Prevotellaceae	GSH-Px	-0.87194576	2.41E-06
Prevotellaceae	IgA	-0.807517949	5.08E-05
Prevotellaceae	T-SOD	-0.953818377	9.00E-10
Prevotellaceae	ROS	0.953977261	8.76E-10
Prevotellaceae	AA	-0.810389247	4.55E-05
Prevotellaceae	PA	-0.907072702	2.08E-07
Prevotellaceae	BA	-0.866175564	3.37E-06
Prevotellaceae	Nrf2	-0.926249343	3.49E-08
Prevotellaceae	HO-1	-0.944498827	3.80E-09
Prevotellaceae	CD19	-0.833654984	1.72E-05
Prevotellaceae	IL-10	-0.91581898	9.71E-08
Prevotellaceae	AMPK	-0.832031217	1.85E-05
Prevotellaceae	IL-4	-0.900934841	3.40E-07
Prevotellaceae	TGF-B	0.876003461	1.89E-06
Prevotellaceae	Inos	0.858188024	5.22E-06
Eubacterium_coprostanoligenes_group	IgM	-0.840840735	1.24E-05
Eubacterium_coprostanoligenes_group	GSR	-0.810477604	4.53E-05
Eubacterium_coprostanoligenes_group	MDA	0.813679292	4.00E-05
Eubacterium_coprostanoligenes_group	Keap1	0.974434551	8.47E-12
Erysipelatoclostridiaceae	Nrf2	-0.819918375	3.11E-05
Erysipelatoclostridiaceae	TGF-B	0.856131446	5.82E-06
Erysipelatoclostridiaceae	iNOS	0.889662623	7.77E-07
Enterococcaceae	IgM	-0.844572326	1.04E-05
Enterococcaceae	Keap1	0.987353424	3.16E-14
Eggerthellaceae	GSH-Px	0.891038582	7.06E-07
Eggerthellaceae	T-SOD	0.851112813	7.53E-06
Eggerthellaceae	ROS	-0.94904994	1.95E-09
Eggerthellaceae	PA	0.878140621	1.66E-06

Eggerthellaceae	BA	0.816194259	3.61E-05
Eggerthellaceae	Nrf2	0.923655303	4.56E-08
Eggerthellaceae	HO-1	0.86318937	3.98E-06
Eggerthellaceae	IL-10	0.85147303	7.39E-06
Eggerthellaceae	IL-4	0.858606369	5.10E-06
Eggerthellaceae	TGF-B	-0.821636212	2.89E-05
Eggerthellaceae	iNOS	-0.829187199	2.10E-05
