# Supplementary figures and images for: Supplementing Ryegrass Ameliorates Commercial Diet-Induced Gut Microbial Dysbiosis-Associated Spleen Dysfunctions by Gut–Microbiota–Spleen Axis
Source: Nutrients. 2024 Mar 5;16(5):747. doi: 10.3390/nu16050747 (PMC10935305; doi:10.3390/nu16050747)

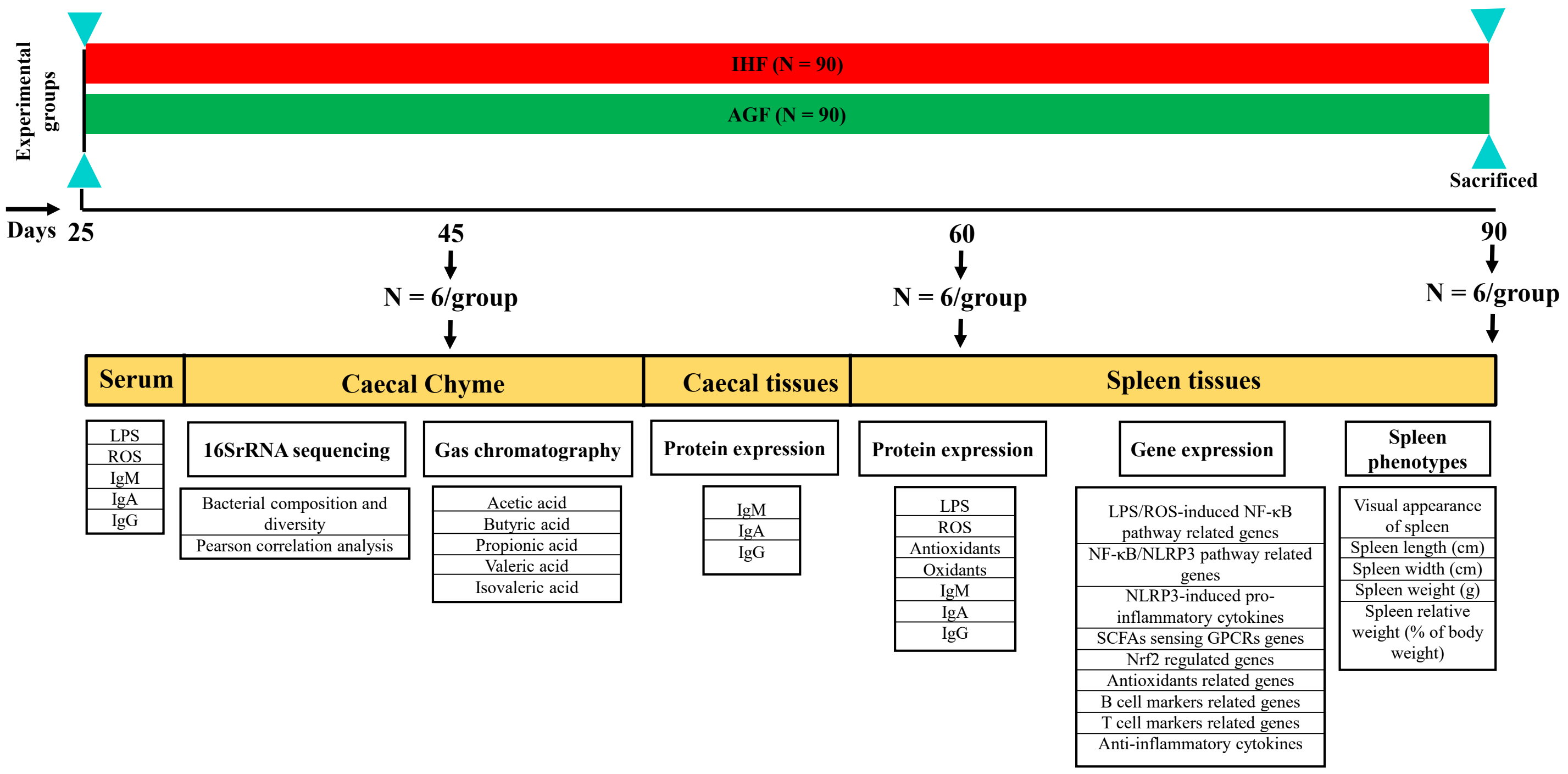

Supplement: Supplementary file 1 [file nutrients-16-00747-s001.zip › nutrients-2844354-supplementary/Figure S1.pdf]

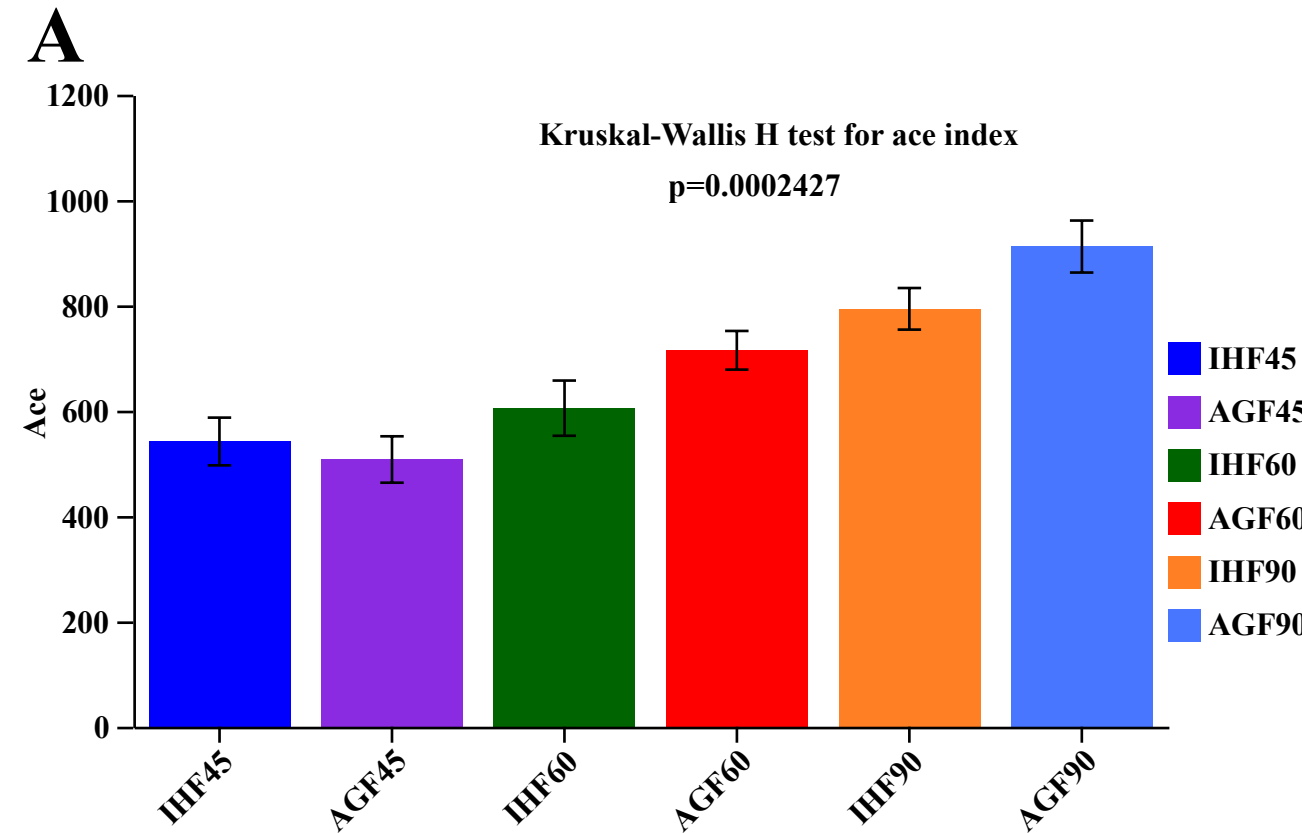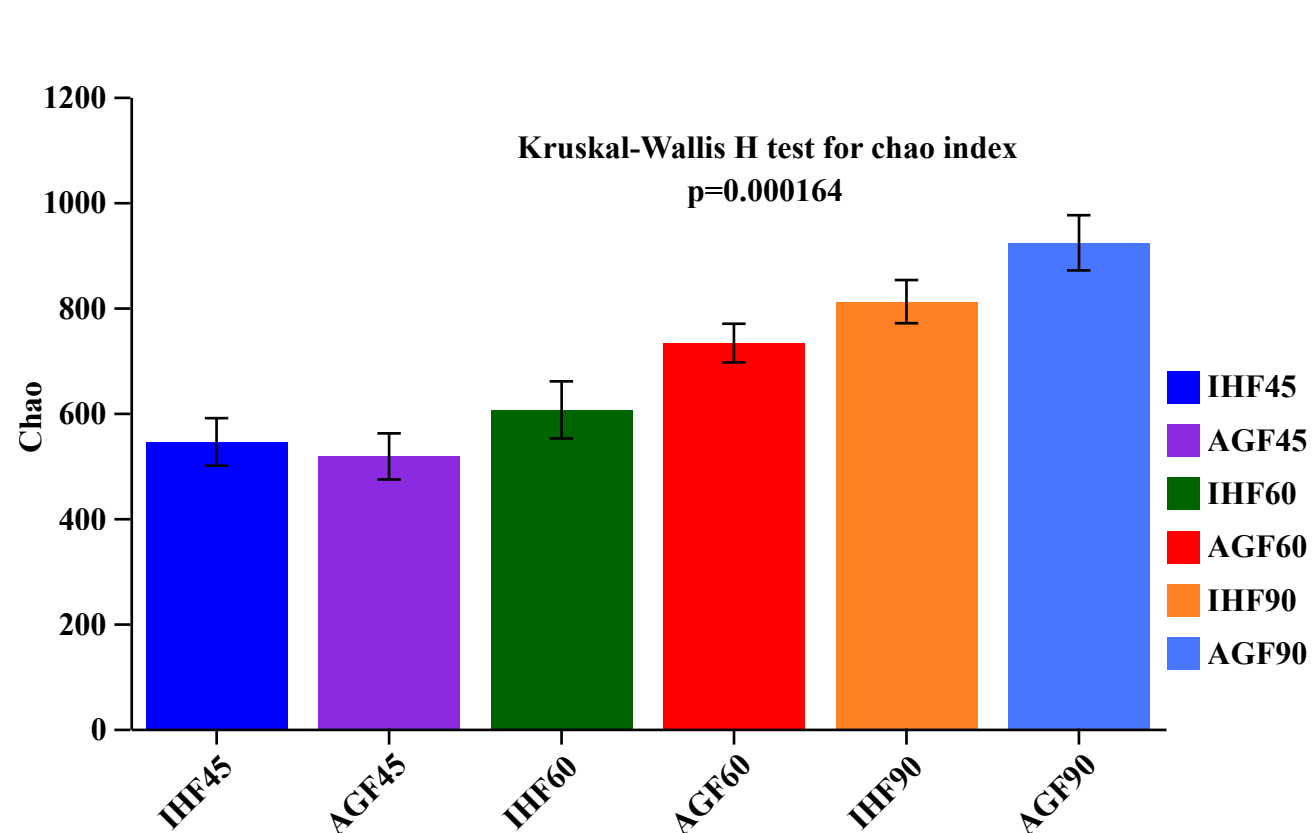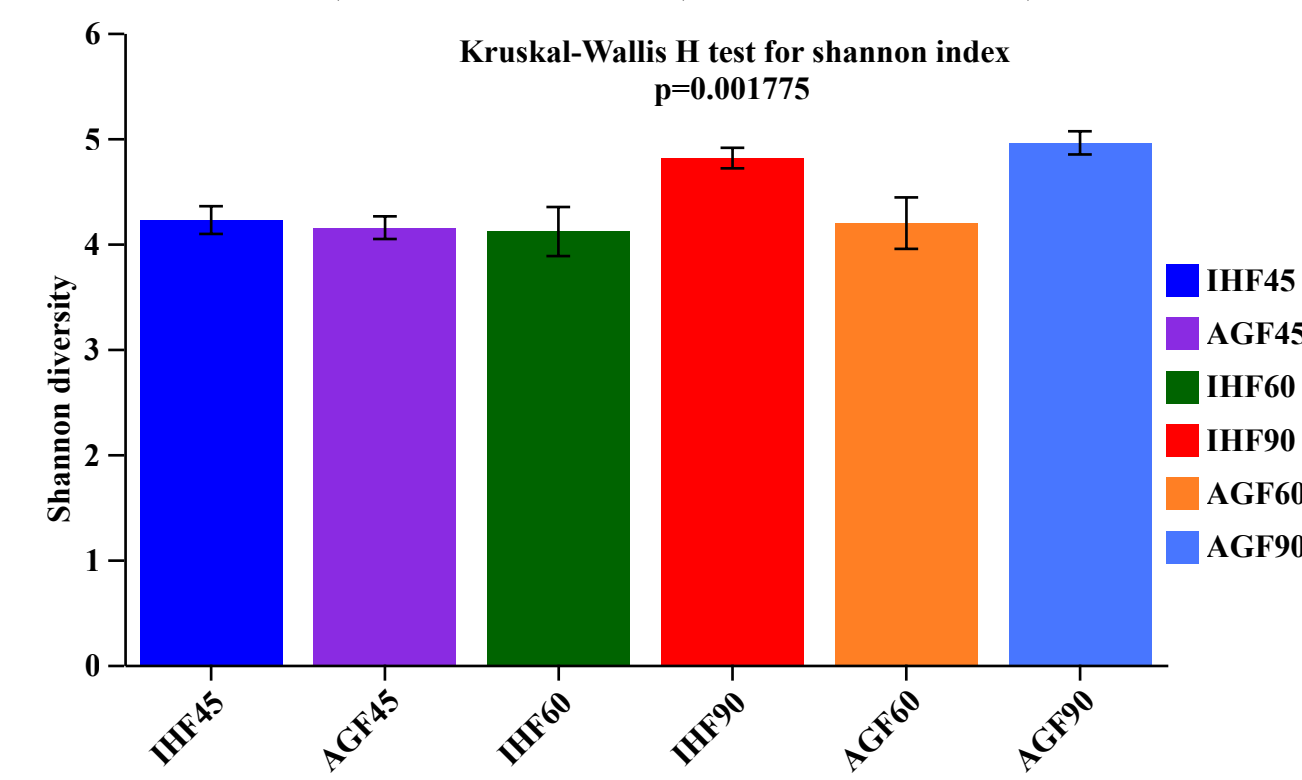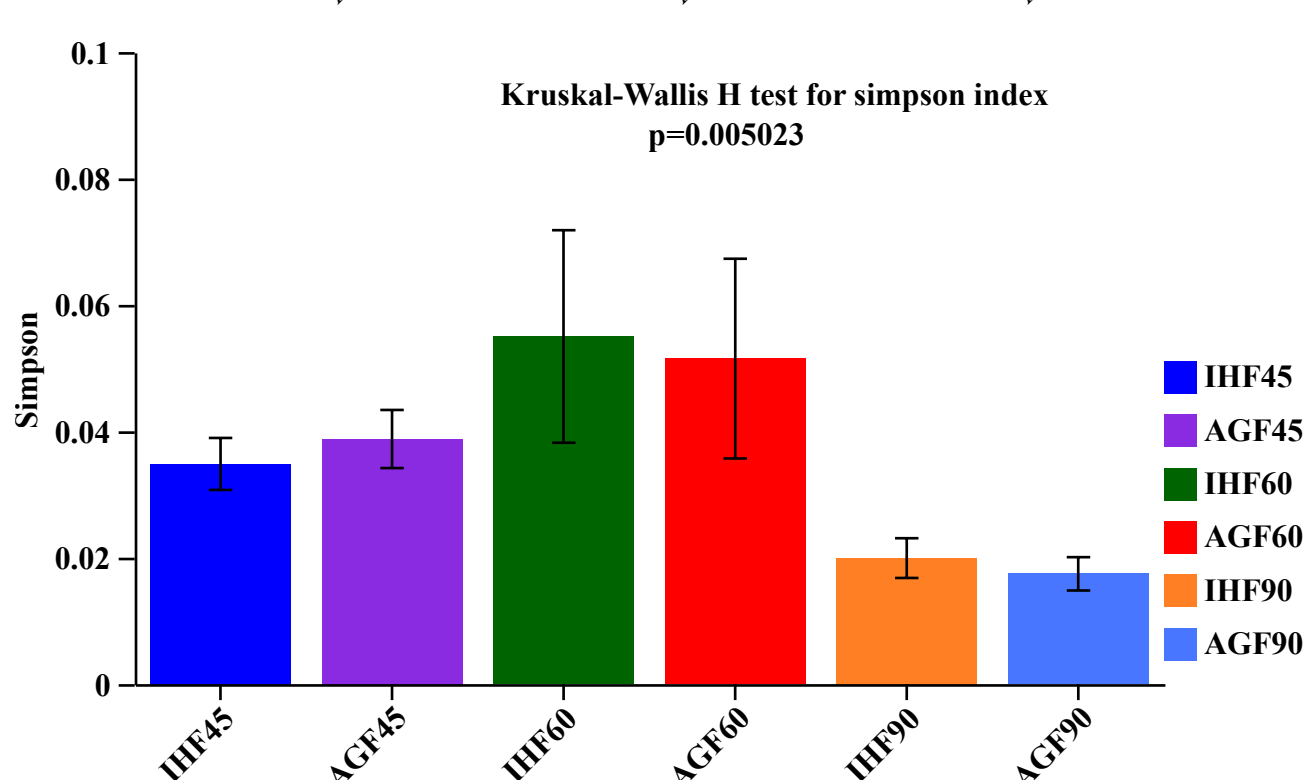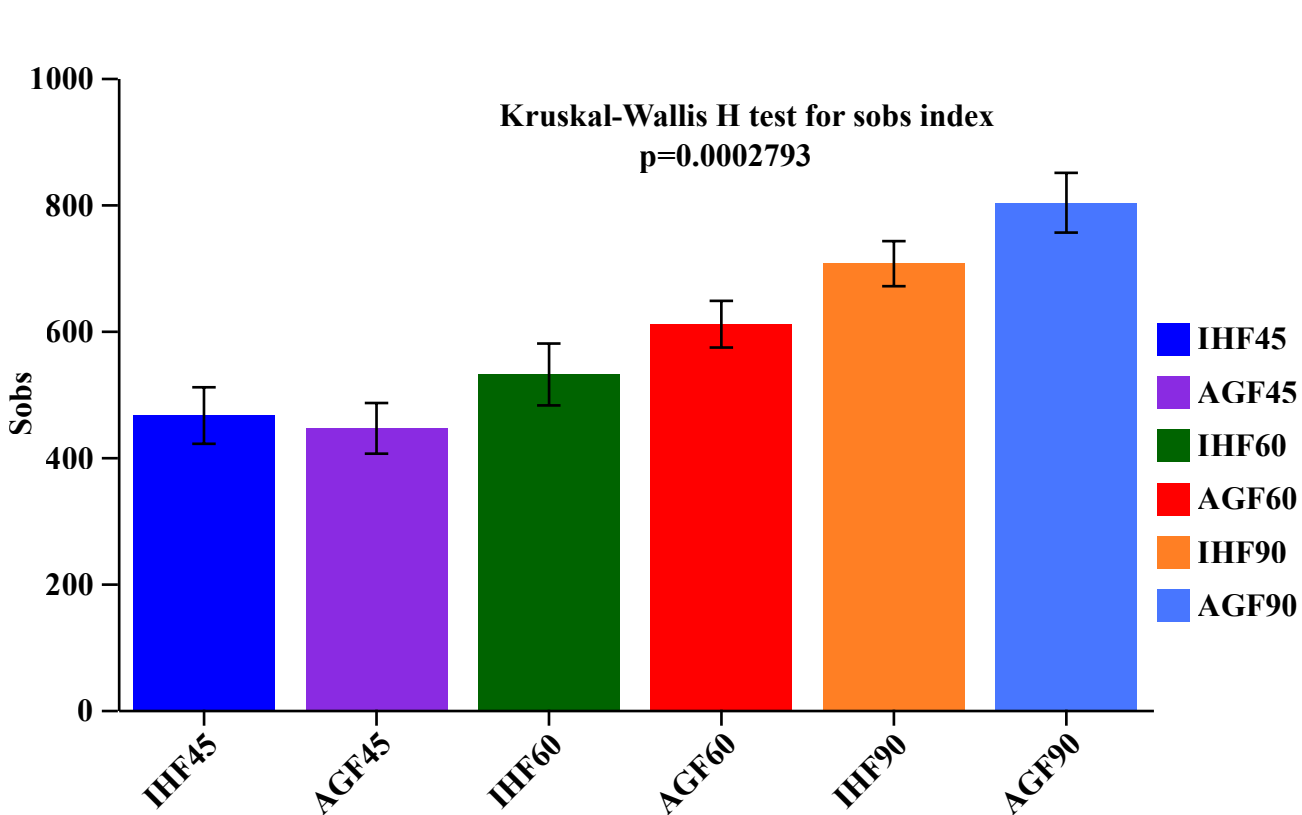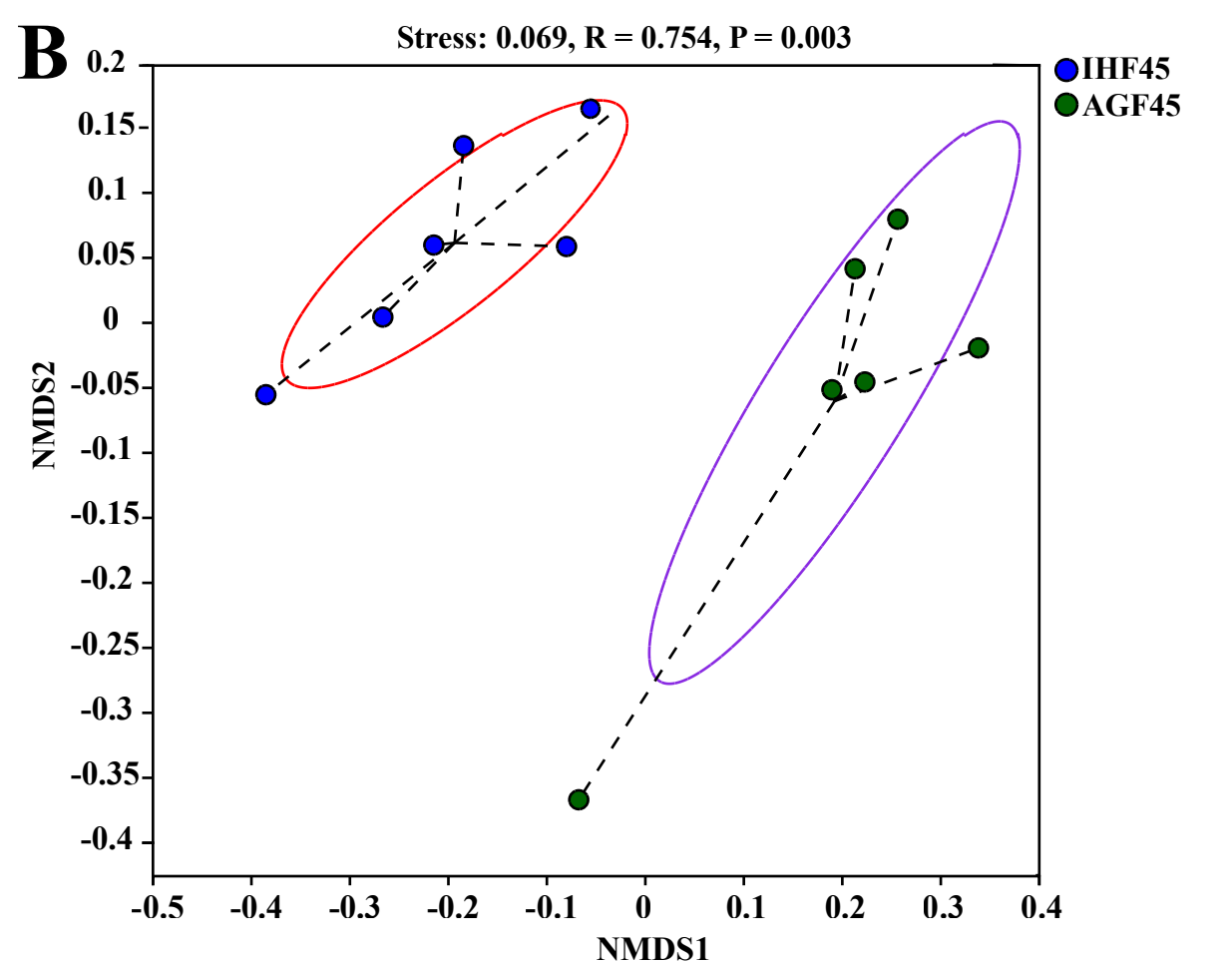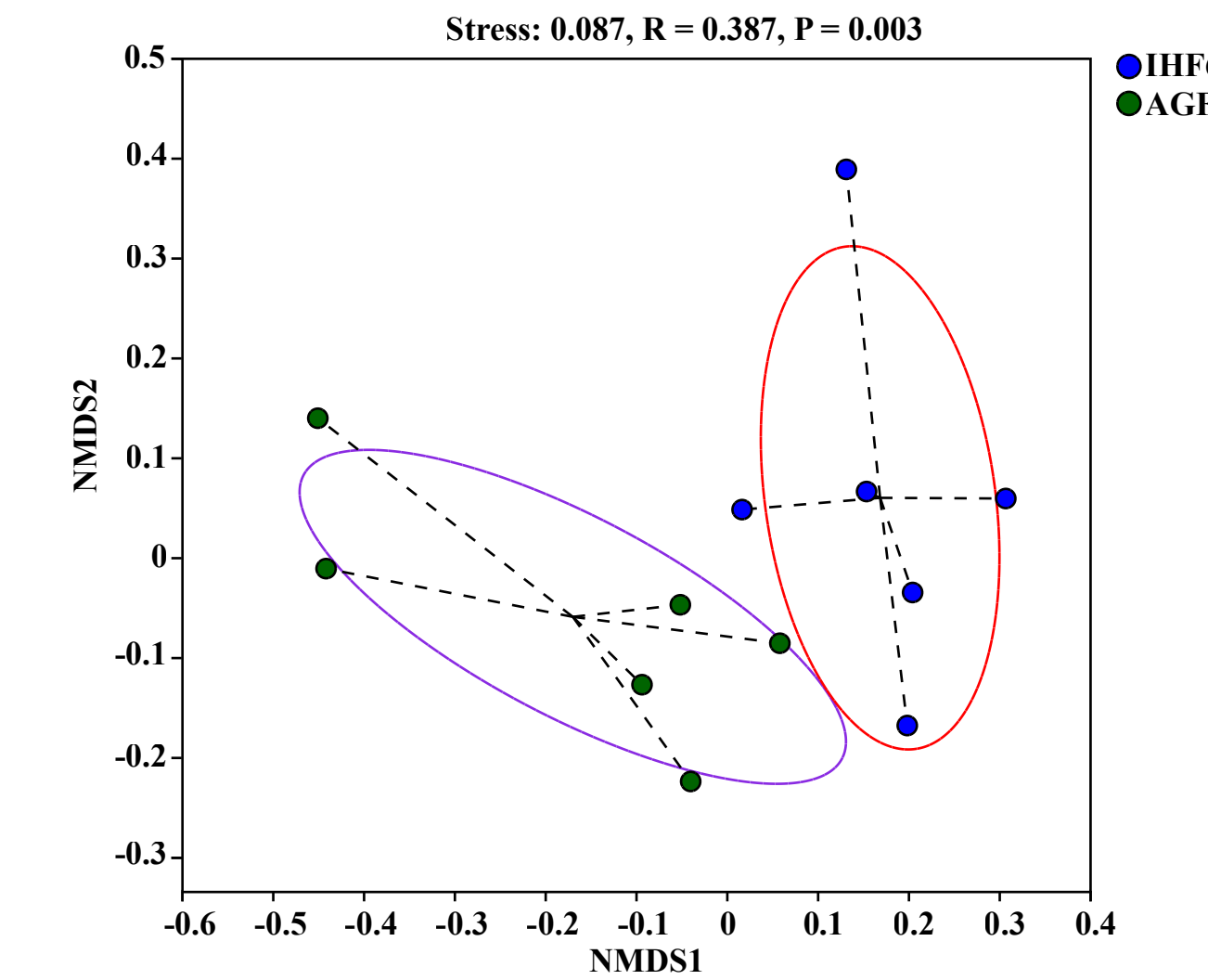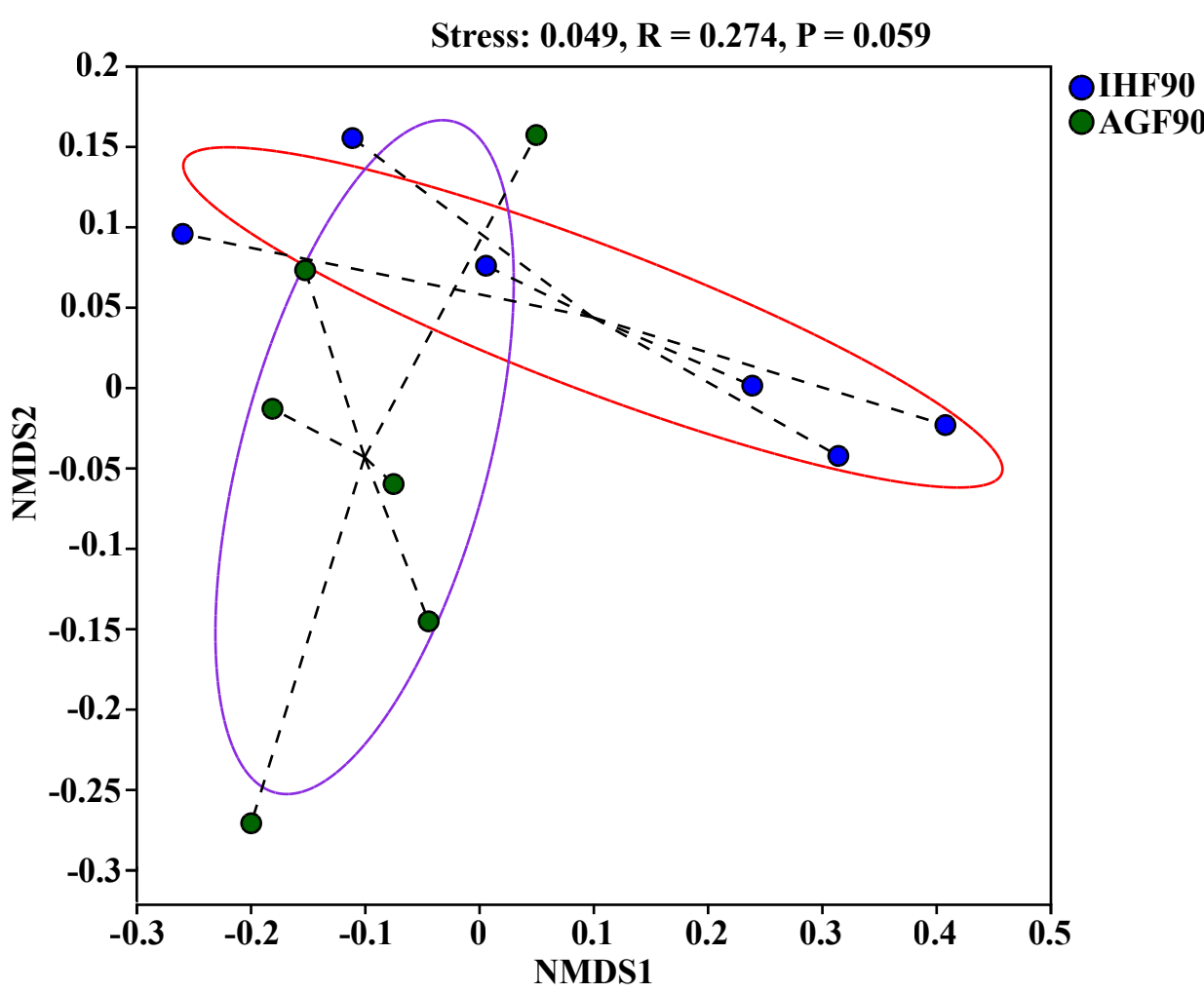

Supplement: Supplementary file 1 [file nutrients-16-00747-s001.zip › nutrients-2844354-supplementary/Figure S2.pdf]

**A**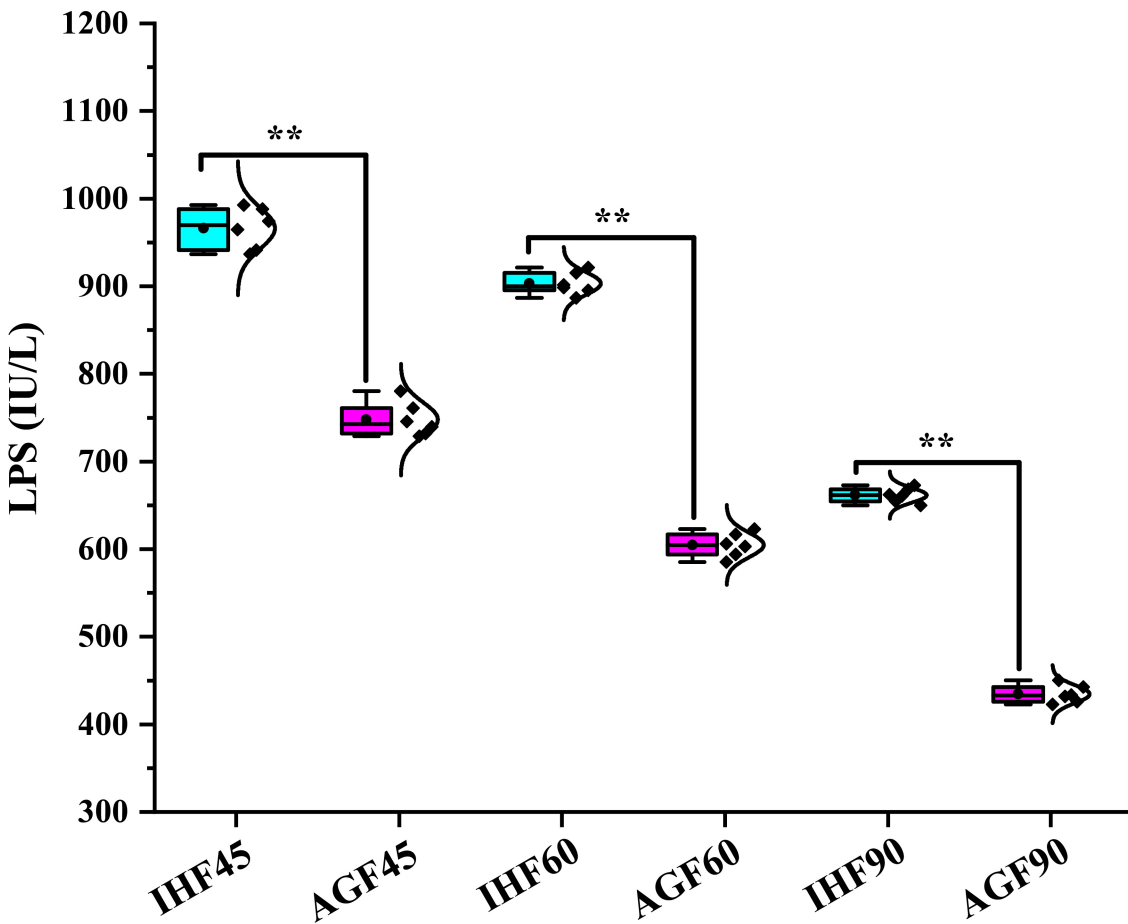**B**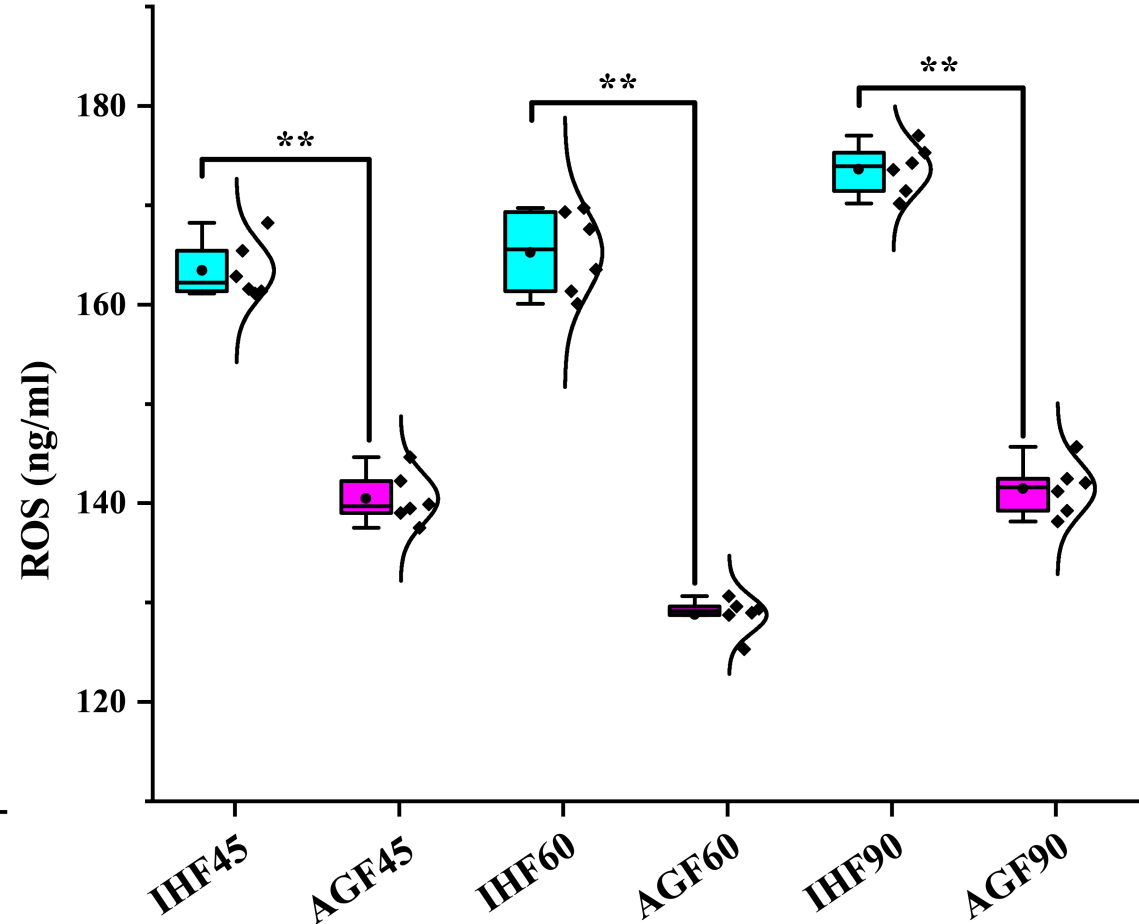

Supplement: Supplementary file 1 [file nutrients-16-00747-s001.zip › nutrients-2844354-supplementary/Figure S3.pdf]

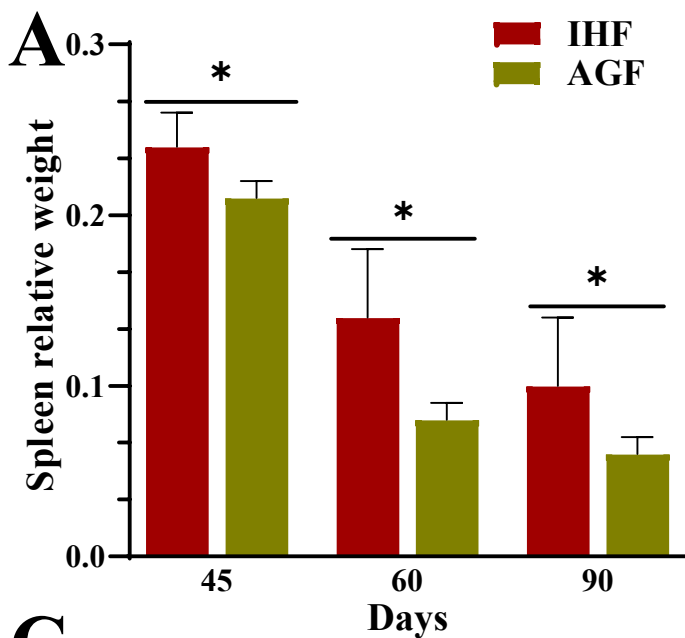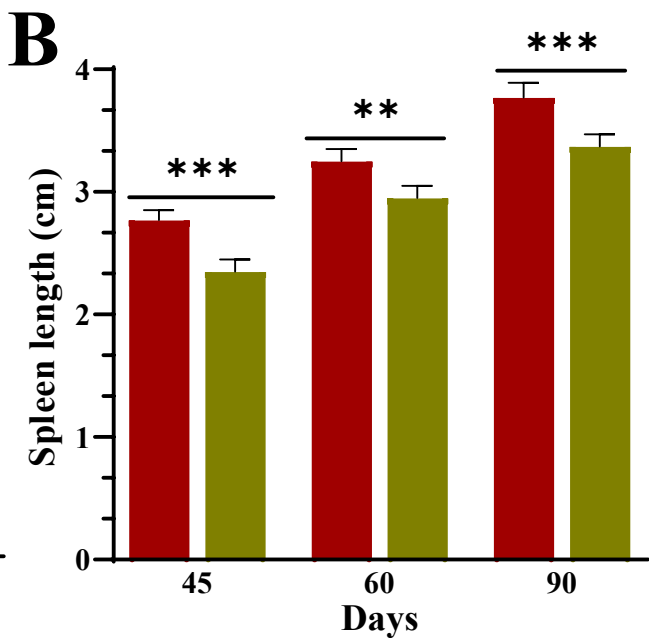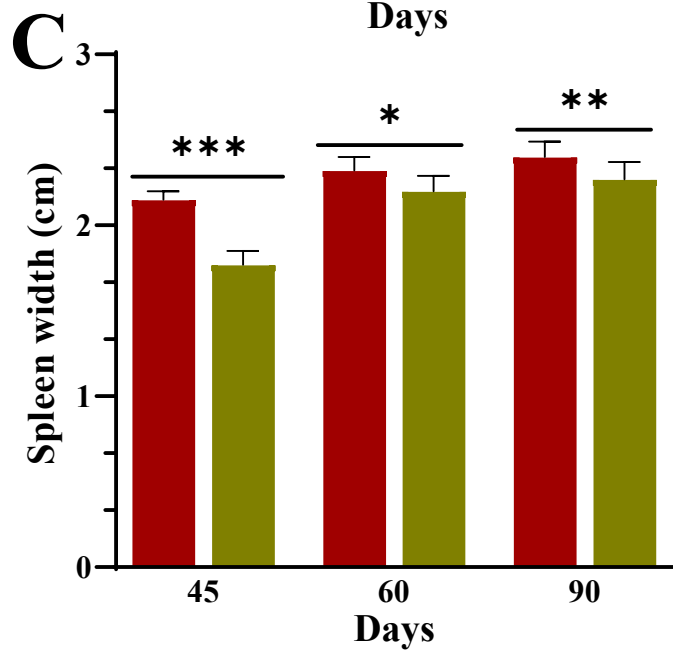

Supplement: Supplementary file 1 [file nutrients-16-00747-s001.zip › nutrients-2844354-supplementary/Figure S4.pdf]

**A****Nrf2 regulated genes**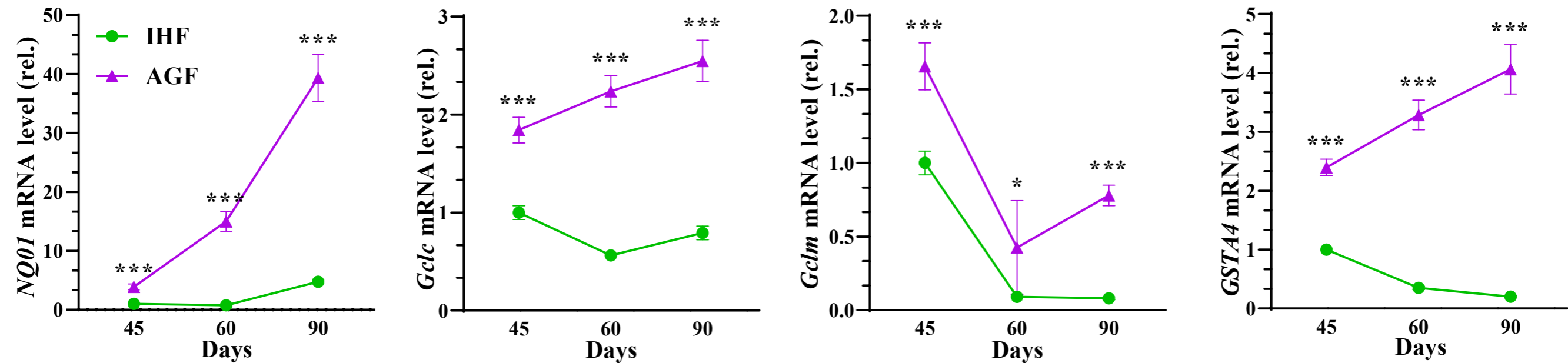**B**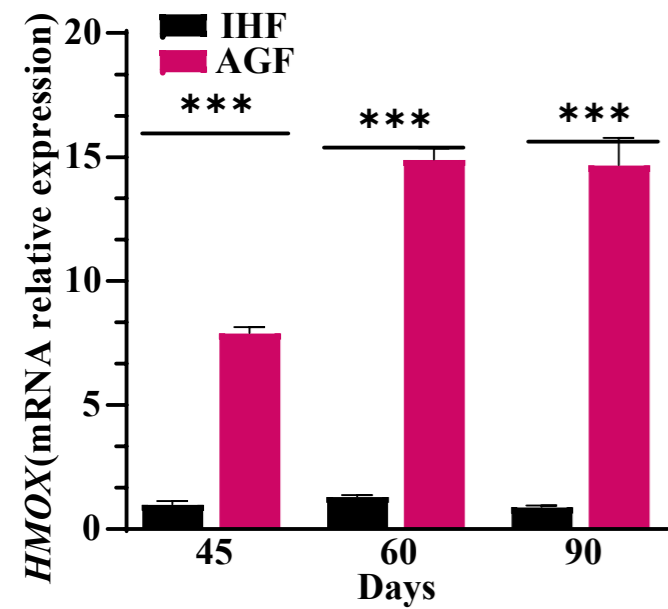**C**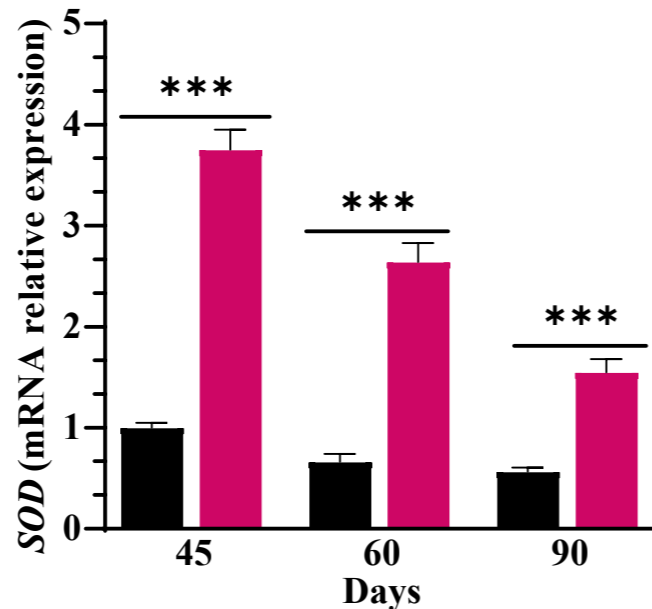**D**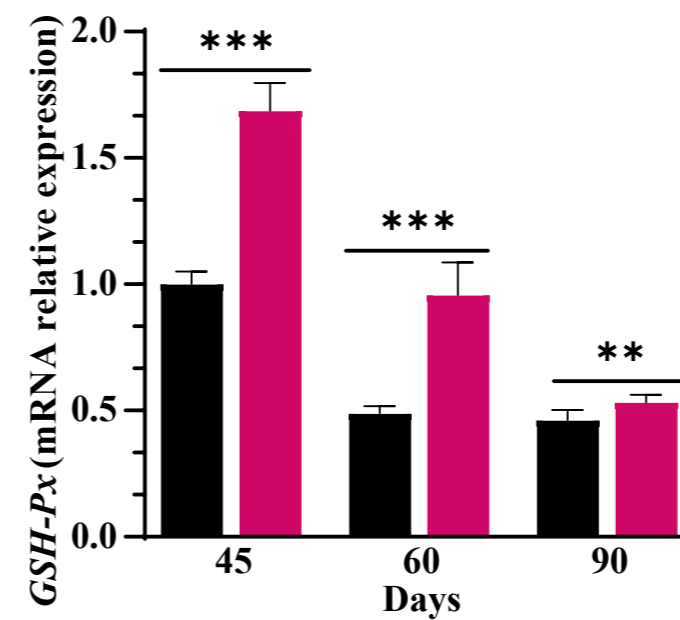**E**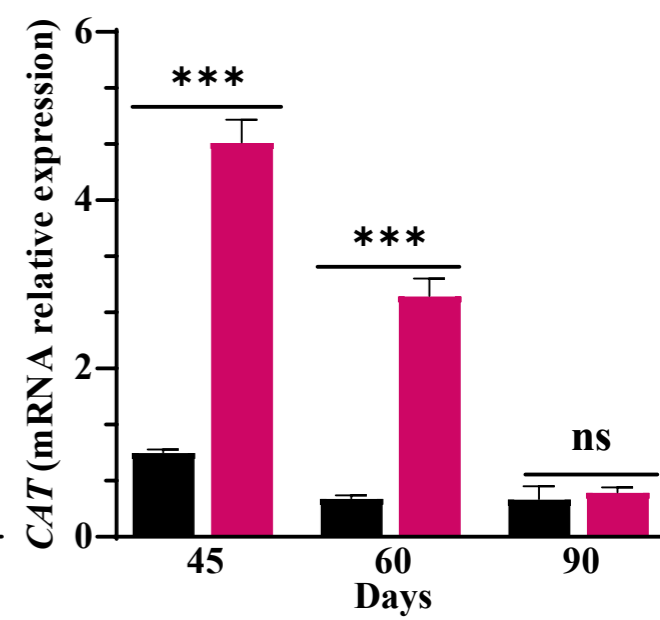

Supplement: Supplementary file 1 [file nutrients-16-00747-s001.zip › nutrients-2844354-supplementary/Figure S5.pdf]

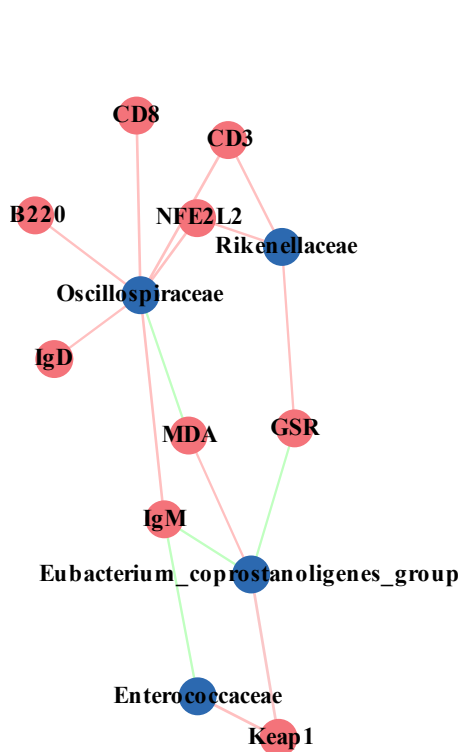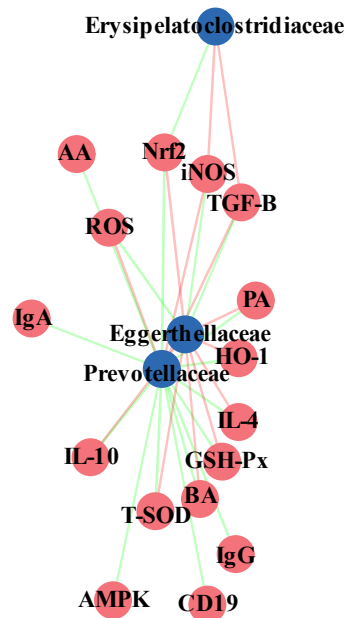

## Relation

— Positive

— Negative

## Spearman coefficient

— 0.70

— 0.99

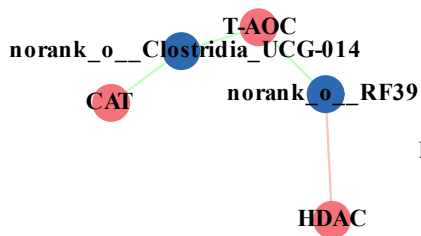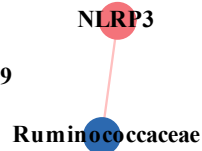

Supplement: Supplementary file 1 [file nutrients-16-00747-s001.zip › nutrients-2844354-supplementary/Figure S6.pdf]
